# Rbm38 Reduces the Transcription Elongation Defect of the *SMEK2* Gene Caused by Splicing Deficiency

**DOI:** 10.3390/ijms21228799

**Published:** 2020-11-20

**Authors:** Shintaro Muraoka, Kazuhiro Fukumura, Megumi Hayashi, Naoyuki Kataoka, Akila Mayeda, Daisuke Kaida

**Affiliations:** 1 Graduate School of Medicine and Pharmaceutical Sciences, University of Toyama, 2630 Sugitani, Toyama 930-0194, Japan; snow_murashin@yahoo.co.jp (S.M.); idenshihatsugen@yahoo.co.jp (M.H.); 2 Division of Gene Expression Mechanism, Institute for Comprehensive Medical Science, Fujita Health University, 1-98 Dengakugakubo, Kutsukake-cho, Toyoake, Aichi 470-1192, Japan; fukumura@fujita-hu.ac.jp (K.F.); mayeda@fujita-hu.ac.jp (A.M.); 3 Department of Animal Resource Sciences, Graduate School of Agricultural and Life Sciences, The University of Tokyo, 1-1-1 Yayoi, Bunkyo-ku, Tokyo 113-8657, Japan; akataoka@g.ecc.u-tokyo.ac.jp

**Keywords:** pre-mRNA splicing, Rbm38, transcription elongation, spliceostatin A

## Abstract

Pre-mRNA splicing is an essential mechanism for ensuring integrity of the transcriptome in eukaryotes. Therefore, splicing deficiency might cause a decrease in functional proteins and the production of nonfunctional, aberrant proteins. To prevent the production of such aberrant proteins, eukaryotic cells have several mRNA quality control mechanisms. In addition to the known mechanisms, we previously found that transcription elongation is attenuated to prevent the accumulation of pre-mRNA under splicing-deficient conditions. However, the detailed molecular mechanism behind the defect in transcription elongation remains unknown. Here, we showed that the RNA binding protein Rbm38 reduced the transcription elongation defect of the *SMEK2* gene caused by splicing deficiency. This reduction was shown to require the N- and C-terminal regions of Rbm38, along with an important role being played by the RNA-recognition motif of Rbm38. These findings advance our understanding of the molecular mechanism of the transcription elongation defect caused by splicing deficiency.

## 1. Introduction

Pre-mRNA splicing is an indispensable mechanism for ensuring accurate gene expression in eukaryotes [1,2,3]. It has been reported that more than 95% of human genes consist of exons and intervening sequences, or introns. Introns are precisely excised, after which the exons are joined by pre-mRNA splicing to produce mature mRNAs. The spliced, mature mRNAs are exported to the cytoplasm and translated into functional proteins. Therefore, this splicing defect often causes the accumulation of pre-mRNAs, which can be exported or leak from the nucleus, before being translated into aberrant proteins [4,5]. To prevent translation from pre-mRNAs, eukaryotic cells have several mRNA quality control mechanisms. When pre-mRNAs accumulate due to splicing deficiency, the nuclear exosome degrades them [6]; otherwise, they are retained in the nucleus [7,8,9]. Pre-mRNAs that are exported or leak from the nucleus are degraded by nonsense-mediated mRNA decay (NMD) in the cytoplasm [10]. These mRNA quality control mechanisms prevent the production of aberrant proteins that are translated from pre-mRNAs. In addition to the aforementioned mRNA quality control mechanisms, we found that transcription elongation is attenuated to prevent mRNA accumulation and translation upon treatment with spliceostatin A (SSA), which is a potent splicing inhibitor that binds to U2 small nuclear ribonucleoprotein particle (snRNP) and inhibits splicing [4,11,12,13]. We previously found that SSA causes gene-specific 3′-end down-regulation (~2800 genes out of 15,800 genes) and the accumulation of RNA polymerase II (Pol II) near the 5′ end of the genes, including *CDK6*, *SMEK2*, and *EGFR* [11,12]. In addition, the phosphorylation level of the C-terminal domain (CTD) of Pol II, which is a hallmark of active transcription, is decreased in SSA-treated cells [11]. These results suggest that SSA treatment leads to a transcription elongation defect on these genes. We postulated that cells detect a splicing defect and stop transcription to prevent pre-mRNA accumulation and pre-mRNA translation, although the detailed molecular mechanism behind this process remains unknown.

RNA binding proteins (RBPs) are key regulators of gene expression in eukaryotes [14,15]. In particular, the binding of RBPs to pre-mRNAs regulates various RNA processing events, including capping, polyadenylation, and splicing. Such RBPs bind to their target RNA through their RNA binding motifs, including the RNA binding domain (RBD, also known as the RNA recognition motif, RRM), heterogeneous nuclear ribonucleoprotein particle (hnRNP) K-homology domain, RGG box, zinc finger domain, and other accessory domains [14,15,16,17]. One of the most well-characterized RNA binding domains is RBD/RRM, which is possessed by 1% of human proteins [18]. RBD/RRM contains two conserved sequences—RNP1 and RNP2—which are indispensable for the RNA binding ability of RBD/RRM [14,19]. Their consensus sequences are K/R-G-F/Y-A/G-F/Y-I/L/V-X-F/Y and I/L/V-F/Y-I/L/V-X-N-L, respectively [20]. Each RBD/RRM has its own sequence preference and typically binds to a region of 2–6 nt in single-stranded RNA [15,18]. In this study, we found that the RBP Rbm38 suppresses the transcription elongation defect caused by splicing deficiency, and that the N- and C-terminal regions and the RBD/RRM domain of Rbm38 are required for this suppression.

## 2. Results

### 2.1. Some RBPs Reduce the Transcription Elongation Defect Caused by Splicing Deficiency

**Figure 1:**
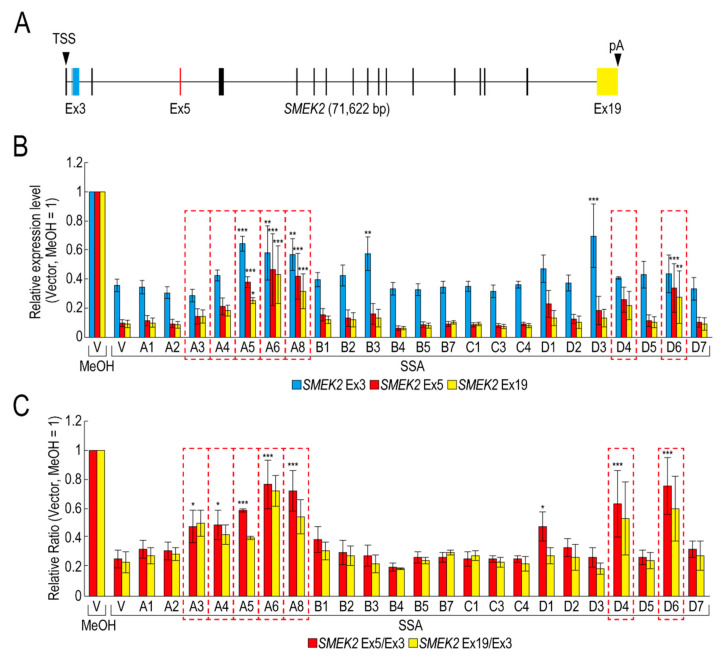
RNA binding proteins (RBPs) reduce the transcription elongation defect caused by splicing deficiency. (**A**) Schematic representation of the *SMEK2* gene. Black arrowheads indicate the transcription start site and the polyA site. Cyan, red, and yellow rectangles are Ex3, Ex5, and Ex19, respectively. (**B**,**C**) HeLa cells were transfected with a vector (V) or the indicated RBP in our library, and then cultured for 48 h after transfection. The cells were treated with MeOH or spliceostatin A (SSA) (10 ng/mL) for 1 h, and then treated with 200 µM 5-EU for an additional 2 h to label nascent RNA. The labeled RNA was analysed by quantitative RT-PCR. Relative expression levels of *SMEK2* Ex3, Ex5, and Ex19 were plotted (**B**). Ratios of the expression levels of Ex5 or Ex19 relative to that of Ex3 (i.e., Ex5/Ex3 and Ex19/Ex3, respectively) were plotted (**C**). Error bars indicate S.D. (*n* = 3). Statistical significance was investigated by one-way ANOVA and Dunnett’s test (*: *p* < 0.05; **: *p* < 0.01; ***: *p* < 0.001). Red dotted line rectangles indicate RBPs that suppressed the transcription elongation defect.

To obtain insight into the molecular mechanism of the transcription elongation defect caused by splicing deficiency, we searched for RBPs that suppress the transcription elongation defect using our RBP library (Table S1). We selected 23 RBPs that were highly expressed in transfected HeLa cells for the screening (Figure S1). Following transfection with RBP plasmids, the transfected cells were treated with SSA, after which the nascent mRNAs were analysed to measure the expression level of *SMEK2* and *CDK6* genes, because we previously found that SSA treatment causes a significant transcription elongation defect of these genes [11,12]. Upon SSA treatment, the expression levels of *SMEK2* Ex3, Ex5, and Ex19 were decreased in the vector-transfected cells (Figure 1A,B, V, SSA). The expression levels of Ex5 and Ex19 decreased much more than that of Ex3, indicating that splicing deficiency causes a transcription elongation defect, consistent with previous reports (Figure 1B) [11,12]. To characterize the transcription elongation defect more clearly, we calculated the ratio of the expression levels of Ex5 or Ex19 to that of Ex3 (i.e., Ex5/Ex3 and Ex19/Ex3, respectively) (Figure 1C). SSA attenuated transcription elongation (Figure 1C, V, MeOH and V, SSA), and A3, A4, A5, A6, A8, D4, and D6 activated transcription elongation more than two-fold compared to the vector plasmid (Figure 1C). It is important to note that none of the RBPs did not affect transcription elongation of the *CDK6* gene (data not shown). In addition, because the level of expression of the RBPs is variable between proteins (Figure S1), we cannot rule out the possibility that RBPs which did not affect transcription elongation in this assay might activate transcription if the RBPs were highly expressed. Of the seven RBPs, A5, A6, and A8 increased the Ex3 expression level (Figure 1B), whereas A3, A4, D4, and D6 did not affect it, suggesting that A5, A6, and A8 activate transcription initiation or stabilize mRNA, in addition to transcription elongation. Interestingly, B3 and D3 increased the *SMEK2* Ex3 expression level, but did not affect the Ex5/Ex3 and Ex19/Ex3 ratios, indicating that these RBPs activate transcription initiation or stabilize mRNA, but do not affect transcription elongation.

We have previously reported that SSA treatment causes the dephosphorylation of Pol II CTD [11]. If these RBPs activate the transcription elongation globally, the phosphorylation status of Pol II CTD should be restored. However, only A3 restored the phosphorylation, whilst the others had a small, if any, effect on the phosphorylation level (Figure S2). Therefore, this result suggests that, other than A3, these RBPs did not affect global transcription, but a limited number of genes, including *SMEK2*.

### 2.2. Rbm38 Binds to SMEK2 Intron 4 and Reduces the Transcription Elongation Defect

**Figure 2:**
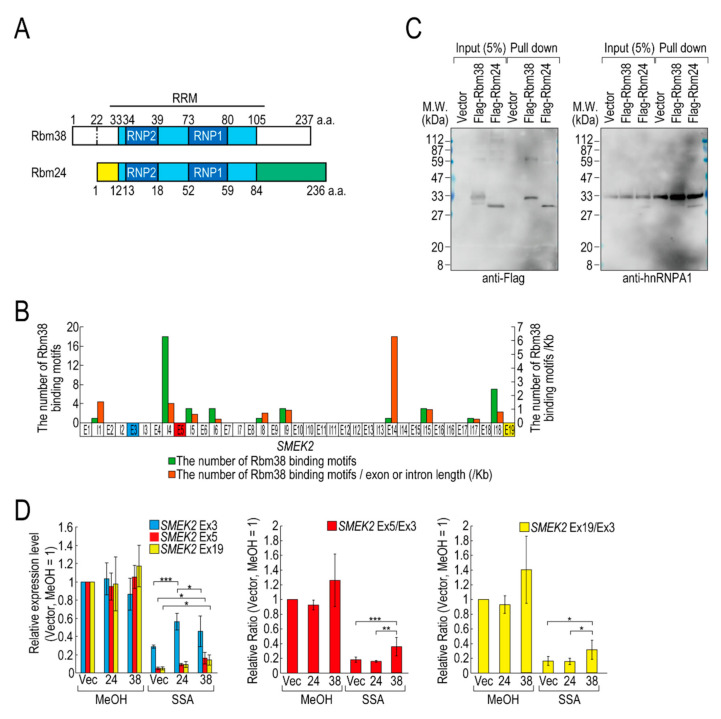
Rbm38, but not Rbm24, suppresses the transcription elongation defect caused by splicing deficiency. (**A**) A schematic of the structure of the indicated RNA binding proteins. (**B**) The distribution of Rbm38 binding motifs in the *SMEK2* gene. The number of Rbm38 binding sites listed in Table S2 was counted. (**C**) Flag-Rbm38 and Flag-Rbm24 were expressed in HEK293T cells and the biotinylated substrate RNA was prepared by in vitro transcription from an SMEK2 plasmid containing intron 4. The substrate RNA was purified using streptavidin beads and co-precipitated proteins were analysed by Western blotting. (**D**) HeLa cells were transfected with a vector (Vec), and Flag-Rbm24 (24) and Flag-Rbm38 (38) plasmids. The transfected cells were treated with SSA and 5-EU, and the labeled RNA was analysed as in Figure 1. Error bars indicate S.D. (*n* = 3). Statistical significance was investigated by one-way ANOVA and Tukey’s test (*: *p* < 0.05; **: *p* < 0.01; ***: *p* < 0.001).

Of the RBPs, we focused on D4 (hereafter Flag-Rbm38) for further experiments for the following two reasons. Flag-Rbm38 reduced the transcription elongation defect, but D3 (hereafter Flag-Rbm24) did not, despite them being highly homologous (Figure 2A) [21]. Therefore, we expected that we would be able to obtain clues to understand the molecular mechanism behind the transcription elongation defect by comparing the effects of the overexpression of Rbm38 and Rbm24. In particular, the RBD/RRMs of the two proteins are almost identical (97% identical), but their N- and C-terminal regions are less homologous (75% and 54% identical, respectively) than the RBD/RRMs. Therefore, we suspected that the N-terminal and/or C-terminal regions of Rbm38 are important for reduction of the transcription elongation defect. The second reason for focusing on Flag-Rbm38 is that putative Rbm38 binding sites, as described in a previous report (Table S2) [22], accumulate in *SMEK2* intron 4 (Figure 2B). Because the expression level of *SMEK2* Ex5 was much lower than that of *SMEK2* Ex3 in SSA-treated cells and the overexpression of Rbm38 recovered the expression level of *SMEK2* Ex5 (Figure 1), transcription elongation appeared to be inhibited between Ex3 and Ex5, and Rbm38 binds to *SMEK2* intron 4 to activate transcription elongation. Therefore, we assumed that we could test whether the RNA binding ability of Rbm38 is important for restoring the attenuated transcription elongation through observing the effects of Flag-Rbm38 overexpression on *SMEK2* transcription.

We investigated whether Rbm38 and Rbm24 bind to *SMEK2* intron 4 by an in vitro binding assay. To this end, we prepared RNA substrates by in vitro transcription using an *SMEK2* intron 4-containing plasmid (Figure S3) and found that both of the proteins bound to the substrate RNA (Figure 2C). We also investigated the binding of hnRNP A1 to the RNA as a positive-control and found that hnRNP A1 bound to the RNA in all samples.

Next, we compared the effects of the overexpression of Rbm38 and Rbm24 on transcription elongation in detail. HeLa cells were transfected with a vector or Flag-RBM38 or Flag-RBM24 plasmid, and then treated with SSA or MeOH (the solvent for SSA) as a control. The overexpression of Rbm38 had a slight effect on the transcription of *SMEK2* in MeOH-treated cells, but the differences were not statistically significant (Figure 2D). With SSA treatment, Rbm38 overexpression reduced the elongation defect, whereas Rbm24 did not, which is consistent with the above results (Figure 1A,B and Figure 2D). Interestingly, however, the transcription elongation defect of *CDK6* and *VEGFA* was not suppressed by Rbm38, suggesting that the suppression is gene-specific (Figure S4A,B), consistent with the result above (Figure S2).

Because splicing deficiency causes a transcription elongation defect, we assumed that transcription elongation should resume if splicing is activated by Rbm38. To test this hypothesis, we evaluated the amount of spliced mRNA, but neither Rbm38 nor Rbm24 suppressed the splicing defect (Figure S4C). Therefore, reduction of the transcription elongation defect is not due to splicing activation by Rbm38.

### 2.3. The N- and C-Terminal Regions of Rbm38 Are Important for Reducing the Transcription Elongation Defect

**Figure 3:**
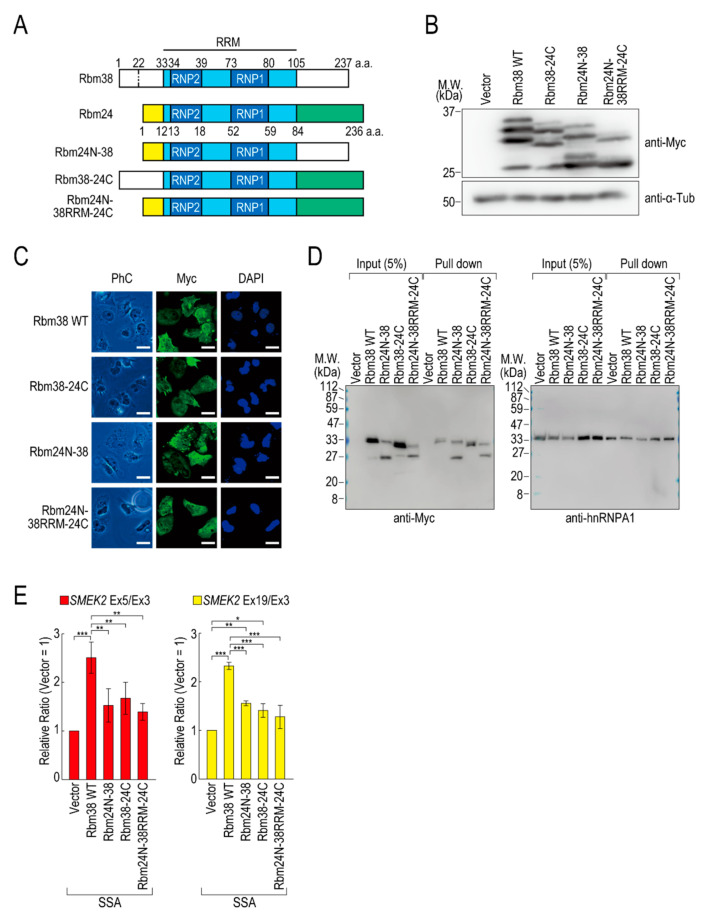
The N- and C-terminal regions of Rbm38 are important for reducing the transcription elongation defect. (**A**) A schematic of the structure of chimeric RNA binding proteins. (**B**,**C**) HeLa cells were transfected with a vector or chimeric plasmids, and then cultured for 48 h after transfection. Protein samples were prepared and analysed by Western blotting (**B**). The subcellular localization of chimeric proteins was analysed by immunofluorescence microscopy (**C**). Bar = 20 µm. (**D**) A biotinylated RNA pull-down assay was performed using chimeric proteins as in Figure 2C. (**E**) HeLa cells were transfected with a vector or chimeric constructs, and then treated with SSA and 5-EU. The labeled RNA was analysed as in Figure 1. Error bars indicate S.D. (*n* = 3). Statistical significance was investigated by one-way ANOVA and Tukey’s test (*: *p* < 0.05; **: *p* < 0.01; ***: *p* < 0.001).

To assess the contributions of the N- and C-terminal regions of Rbm38 to the reduction, we constructed N- and/or C-terminal deletion mutants of Rbm38 in the pcDNA 3.1/*myc*-His vector. We confirmed that Myc-Rbm38, as well as Flag-Rbm38, reduced the transcription elongation defect, with an even slightly higher efficiency than Flag-Rbm38 (Figure S5A). Next, we constructed the N- or C-terminal deletion mutants of Rbm38 with the pcDNA 3.1/*myc*-His backbone (Figure S5B). The expression levels of the N- and C-terminal deletion mutant (Rbm38 ΔN + C) and the C-terminal deletion mutant (Rbm38 ΔC) were much lower than that of the wild type (Rbm38 WT), although the N-terminal deletion mutant (Rbm38 ΔN) was highly expressed (Figure S5C). We assessed the RNA binding ability of the deletion mutants and found that none of them bound to the substrate RNA (Figure S5D). To examine the unique role of the N- and C-terminal regions of Rbm38, we constructed three chimeric constructs in which the C-terminal region of Rbm38 is replaced by that of Rbm24, the N-terminal region of Rbm38 is replaced by that of Rbm24, and both the N- and C-terminal regions of Rbm38 are replaced by those of Rbm24 (Figure 3A). We evaluated the expression levels of these chimeric constructs and found that all mutants were highly expressed to the same extent as the Rbm38 WT (Figure 3B). We observed multiple bands of Rbm38 WT and mutants and found that the slowest migrating bands disappeared after phosphatase treatment, but the other bands did not (Figure S6). This result suggests that the slowest migrating bands are phosphorylated forms of Rbm38 WT and mutants, but the other bands are full-length and presumably truncated forms of the proteins. However, the truncation was even observed in the Rbm38 WT-expressing cells, and did not appear to affect the reducing activity of Rbm38. Next, we examined the subcellular localization of the chimeric proteins. As expected, all of them localized in both the nucleus and cytoplasm, as in the case for the wild type (Figure 3C), which is consistent with a previous report [23]. Then, we investigated the RNA binding ability of the chimeric proteins. The Rbm38 WT and all chimeric proteins bound to the RNA substrates (Figure 3D). Finally, we performed a quantitative RT-PCR experiment to investigate the effect of the chimeric proteins on the transcription elongation. The chimeric proteins did not affect the transcription elongation defect, suggesting that both the N- and C-terminal regions of Rbm38 are important for reducing the transcription elongation defect (Figure 3E and Figure S7A).

### 2.4. The RNA Binding Domain of Rbm38 Is Important for Reducing the Transcription Elongation Defect

**Figure 4:**
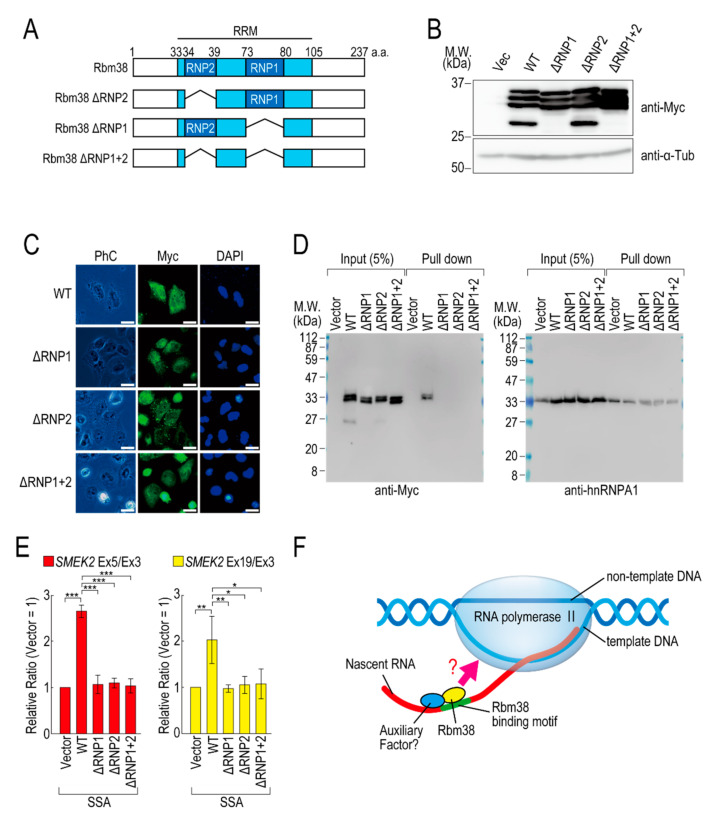
RNA binding motif of Rbm38 is important for suppressing the transcription elongation defect. (**A**) A schematic of the structure of the deleted RNA binding proteins. (**B**,**C**) HeLa cells were transfected with a vector or RNP deletion mutants, and then cultured for 48 h. Protein samples were prepared and analysed by Western blotting (**B**). The subcellular localization of the RNP deletion mutants was analysed by immunofluorescence microscopy (**C**). Bar = 20 µm. (**D**) A biotinylated RNA pull-down assay was performed using RNP deletion mutants as in Figure 2C. (**E**) HeLa cells were transfected with a vector or RNP deletion mutants, and then treated with SSA and 5-EU. The labeled RNA was analysed as in Figure 1. Error bars indicate S.D. (*n* = 3). Statistical significance was investigated by one-way ANOVA and Tukey’s test (*: *p* < 0.05; **: *p* < 0.01; ***: *p* < 0.001). (**F**) A schematic model of the mechanism by which Rbm38 suppresses the transcription defect caused by splicing deficiency. The N- and C-terminal domains and RBD/RRM of Rbm38 are required for the suppression. However, it is still unknown whether the RNA binding ability of Rbm38 is required for the suppression. Furthermore, an auxiliary factor might be involved in the suppression.

To investigate whether the RNA binding ability of Rbm38 is required for reduction of the transcription elongation defect, we constructed RBD/RRM mutants of Rbm38 (Figure 4A). Because RNP1 and RNP2 in the RBD/RRM of Rbm38 are critical for the RNA binding ability [19], we constructed deletion mutants of RNP1 and/or RNP2. All of the deletion mutants were highly expressed in the transfected cells (Figure 4B) and localized in both the nucleus and cytoplasm, as in the case for Rbm38 WT (Figure 4C). Presumably owing to the deletion of RBD/RRM, these proteins could not bind to RNA substrates (Figure 4D). These deletion mutant proteins did not affect the transcription elongation defect either (Figure 4E and Figure S7B), suggesting that the RBD/RRM of Rbm38 is required for reduction of the transcription elongation defect.

## 3. Discussion

We previously found that splicing deficiency causes a transcription elongation defect, although the detailed molecular mechanism remained unknown [11,12]. Here, we showed that Rbm38 reduced the transcription elongation defect caused by splicing deficiency. Rbm38 is an RNA binding protein that regulates mRNA stability, translation activity, alternative splicing, and miRNA accessibility by binding to its target mRNAs via its RBD/RRM domain [19,22,23,24,25,26,27,28,29]. This study demonstrated that the RBD/RRM domain was also indispensable for reduction of the transcription elongation defect. Splicing deficiency caused a transcription elongation defect between exons 3 and 5 of *SMEK2*, and Rbm38 reduced this defect. Interestingly, there are multiple Rbm38 binding sequences in intron 4 of the *SMEK2* gene and we confirmed Rbm38 actually bound to the sequence. Rbm38 had a slight effect on the transcription level of the *SMEK2* gene under normal conditions, presumably because it has less chance of binding to intron 4 of *SMEK2* to activate transcription under normal conditions because splicing leads to the rapid excision of introns. These results suggest that Rbm38 binds to nascent RNA containing Rbm38 binding sites to activate stalled RNA polymerase II (Figure 4F). However, Rbm38 did not affect transcription elongation of the *CDK6* and *VEGFA* genes, although these two genes have several Rbm38 binding sequences. Therefore, Rbm38 binding might be required, but not sufficient, for reduction of the defect, and another cofactor that binds in the vicinity of Rbm38 binding sites might be required for the reduction (Figure 4F). Indeed, in some cases, Rbm38 and its homologue SUP-12 recognize their binding sites in cooperation with other proteins [19,30]. Considering that the RBD/RRM domain is indispensable for reduction of the transcription elongation defect, another interpretation of the results is that RBD/RRM functions as a platform for protein–protein interactions, but not for RNA–protein interactions. Because Rbm38 binds to HuR via its RBD/RRM domain [19], it is also possible that Rbm38 binds to a partner cofactor via its RBD/RRM domain to activate transcription cooperatively.

For reduction of the transcription elongation defect by Rbm38, the N-terminal and C-terminal regions of Rbm38 are also required. The C-terminal region is important for the Rbm38 binding to HuR and eIF4E [19,28]. In addition, the C-terminal region is required for cell cycle arrest and the destabilization of p73, although its binding partner is unknown [23,27]. Therefore, this region is supposed to bind to its binding protein to exert its function. We predict that Rbm38 might recruit a transcription elongation activator via its C-terminal region in the vicinity of RNA polymerase II to activate transcription. However, because Rbm38 modulates gene expression [19,22,23,24,25,26,27,28,29], it is also possible that Rbm38 upregulates the expression of a transcription elongation activator or downregulates the expression of a transcription repressor via its C-terminal region.

In this study, Rbm24 appeared to activate transcription initiation or stabilize several mRNAs, but unlike Rbm38, it did not activate transcription elongation. Therefore, Rbm24 might bind to another set of proteins to exert its functions, presumably through its C-terminal region. In contrast, because both Rbm24 and Rbm38 inhibit the translation of p53, destabilize p63 mRNA, and stabilize p21 mRNAs [19,23,28,29,31,32,33], some of the binding proteins might be common to the two proteins. Rbm24 was reported to bind to U1 snRNP and polyadenylation factors [34,35], whereas the binding between Rbm38 and these splicing and polyadenylation factors has not yet been reported, suggesting that Rbm24- and Rbm38-specific binding proteins contribute to transcription initiation or mRNA stability, and transcription elongation, respectively. We will address how these unique C-terminal regions of the two proteins affect gene expression in future studies.

The N-terminal region is very short and its function is not well-known. We found that deletion of this region of Rbm38 results in loss of the binding capacity for at least the substrate RNA used in this study. Therefore, the N-terminal region might be important for RNA binding. However, because only the RBD/RRM domain of Rbm38 can bind to GU- and U-repeats [36], the N-terminal region might be required for RNA binding in a context-dependent manner. Further study is required to understand the physiological functions of the N-terminal region.

In summary, we found that Rbm38 reduces the transcription elongation defect caused by splicing deficiency. For this reduction, the N- and C-terminal domains and RBD/RRM domain are important. These findings should boost our understanding of the mechanisms of the transcription elongation defect caused by splicing deficiency, which would contribute to protecting the integrity of the transcriptome and cellular functions.

## 4. Materials and Methods

### 4.1. Cell Culture and Reagents

HeLa S3 and HEK293 cells were cultured in Dulbecco’s modified Eagle’s medium (FUJIFILIM Wako Pure Chemical Corporation, Osaka, Japan) containing 10% heat-inactivated fetal bovine serum (Thermo Fisher Scientific, Waltham, MA, USA). Cells were maintained in 5% CO_2_ at 37 °C. SSA was a kind gift from Dr Minoru Yoshida (RIKEN, Saitama, Japan).

### 4.2. RNA Preparation and Quantitative RT-PCR

Plasmid transfection was performed using Lipofectamine 3000 Reagent (Thermo Fisher Scientific), in accordance with the manufacturer’s instructions. Nascent RNA was purified using the Click-iT Nascent RNA Capture Kit (Thermo Fisher Scientific). Briefly, after plasmid transfection, transfected cells were cultured for 48 h, treated with MeOH or SSA (10 ng/mL) for 1 h, and then treated with 5-EU (200 µM) for an additional 2 h to label nascent RNAs. Total RNAs were extracted from cultured cells using TRIzol Reagent (Thermo Fisher Scientific). Labeled RNA was biotinylated by the click reaction, and the biotinylated RNA was purified using streptavidin beads. For quantitative RT-PCR, cDNA was synthesized using the SuperScript VILO cDNA Synthesis Kit (Thermo Fisher Scientific). Quantitative RT-PCR and relative quantification analyses were performed with the MX3000P system (Agilent, Santa Clara, CA, USA) using SYBR Green dye chemistry. All primers are listed in Table S3.

### 4.3. Antibodies

Mouse monoclonal anti-α-tubulin antibody (T6199) and mouse monoclonal anti-Pol II antibody (ARNA-3) were purchased from Sigma-Aldrich (St. Louis, MO, USA). Mouse monoclonal anti-DDDDK (Flag) antibody (FLA-1) and mouse monoclonal anti-Myc antibody (My3) were purchased from Medical and Biological Laboratories (Nagoya, Japan). Mouse monoclonal anti-hnRNP A1 antibody (4B10) was purchased from Santa Cruz Biotechnology (Santa Cruz, CA, USA). HRP-conjugated anti-mouse IgG and anti-rabbit IgG secondary antibodies were purchased from GE Healthcare (Chicago, IL, USA). Secondary antibodies conjugated to Alexa 488-conjugated anti-mouse secondary antibody (#A11001) were purchased from Thermo Fisher Scientific.

### 4.4. Plasmid Construction

To construct the Myc-tagged Rbm38 WT plasmid, the open reading frame (ORF) of *RBM38* was amplified by PCR from the Flag-Rbm38 (D4) plasmid using the mRBM38F and mRBM38R primers. To construct the Rbm38 ∆N and Rbm38 ∆C plasmids, DNA fragments of mRBM38∆N and mRBM38∆C were amplified by PCR from the Flag-Rbm38 (D4) plasmid using the mRBM38∆NF and mRBM38R primers, and mRBM38F and mRBM38∆CR primers, respectively. The PCR products were digested with EcoRI and XhoI, and subcloned into the pcDNA™ 3.1/*myc*-His A vector plasmid (Thermo Fisher Scientific). To construct Rbm38 ∆N+C plasmid, the Rbm38 ∆N and Rbm38 ∆C plasmids were digested with ClaI and XhoI; then, the 3′ half of the Rbm38 ∆N plasmid was replaced by the 3′ half of the Rbm38 ∆C plasmid.

To construct the Rbm24N-38 plasmid, the DNA fragment of *RBM24N-38* was amplified by PCR from the Flag-Rbm38 plasmid using the Chimera1F and mRBM38R primers. The PCR product was digested with EcoRI and XhoI, and subcloned into the pcDNA™ 3.1/*myc*-His A vector plasmid. To construct the Rbm38-24C plasmid, the 5′ half of the *RBM38* ORF and the 3′ half of the *RBM24* ORF were amplified by PCR from the Flag-Rbm38 plasmid and Flag-Rbm24 plasmid, respectively. For amplification of the 5′ half of the *RBM38* ORF, the mRBM38F and Chimera2R primers were used. For amplification of the 3′ half of the *RBM24* ORF, the Chimera3F and mRBM24R primers were used. The second-step PCR was performed using the 5′ half of the *RBM38* ORF and the 3′ half of the *RBM24* ORF as templates with the mRBM38F and mRBM24R primers. The second-step PCR product was digested with EcoRI and XhoI, and subcloned into the pcDNA™ 3.1/*myc*-His A vector plasmid. To construct Rbm24N-38RRM-24C, the Rbm24N-38 and Rbm38-24C plasmids were digested with ClaI and XhoI; then, the 3′ half of the Rbm24N-38 plasmid was replaced by the 3′ half of the Rbm38-24C plasmid.

To construct the Rbm38 ∆RNP1 plasmid, the 5′ half of the *RBM38* fragment and the 3′ half of the *RBM38* fragment were amplified by PCR from the Flag-Rbm38 plasmid using mRBM38F and ∆RNP1R primers and ∆RNP1F and mRBM38R primers, respectively. The second-step PCR was performed using the 5′ half of the *RBM38* ORF and the 3′ half of the *RBM38* ORF as templates with mRBM38F and mRBM38R primers. To construct the Rbm38 ∆RNP2 plasmid, another 5′ half of the *RBM38* fragment and another 3′ half of the *RBM38* fragment were amplified by PCR from the Flag-Rbm38 plasmid, using the mRBM38F and ∆RNP2R primers, and ∆RNP2F and mRBM38R primers, respectively. The second-step PCR was performed using the 5′ half of the *RBM38* ORF and the 3′ half of the *RBM38* ORF as templates with the mRBM38F and mRBM38R primers. The second-step PCR products were digested with EcoRI and XhoI, and subcloned into the pcDNA™ 3.1/*myc*-His A vector plasmid. To construct Rbm38 ∆RNP1+2, Rbm38 ∆RNP1 and Rbm38 ∆RNP2 plasmids were digested with ScaI; then, the 3′ half of the Rbm38 ∆RNP2 plasmid was replaced by the 3′ half of the Rbm38 ∆RNP1 plasmid.

To construct the SMEK2 intron 4 plasmid, the DNA fragment of part of intron 4 of *SMEK2* was amplified by PCR from HeLa genomic DNA using SMEK2 int4 cloning for EcoRI and SMEK2 int4 cloning rev XhoI-3 primers. The PCR product was digested with EcoRI and XhoI and subcloned into the pcDNA™ 3.1/*myc*-His A vector plasmid. All primers for plasmid construction are listed in Table S3. All plasmids used in this study are listed in Table S4.

### 4.5. Western Blotting

Plasmid transfection was performed using Lipofectamine 3000 Reagent (Thermo Fisher Scientific), in accordance with the manufacturer’s instructions. Sample preparation and Western blotting were performed as described previously [11]. Briefly, cells were directly lysed on plates with 1× SDS-PAGE sample buffer. Proteins were then separated by SDS-PAGE. After electrophoresis, the proteins were transferred onto a PVDF membrane by electroblotting. Following incubation of the membrane with primary and secondary antibodies using standard techniques, protein bands were detected using a NOVEX ECL Chemiluminescent Substrate Reagent Kit (Thermo Fisher Scientific) on an ImageQuant LAS 4000mini (GE Healthcare).

### 4.6. Biotinylated RNA Pull-Down Assays

HEK293 cells were transfected with Rbm38-Myc plasmids using Lipofectamine 2000 reagent (Thermo Fisher Scientific). At 48 h post-transfection, cells were harvested, washed with cold PBS buffer, suspended in Buffer D (20 mM HEPES (pH 7.9), 50 mM KCl, 0.2 mM EDTA, and 20% glycerol), sonicated for 20 s, and centrifuged (13,000× *g*, 10 min, 4 °C) to remove debris. Biotin-labeled *SMEK2* intron 4 was transcribed with the SMEK2 intron 4 plasmid and the MEGAscript T7 transcription kit (Thermo Fisher Scientific), in accordance with the manufacturer’s instructions. The biotinylated substrate mRNA (10 pmol) was immobilized with 5 µL of Dynabeads MyOne StreptavidinT1 magnetic beads (Thermo Fisher Scientific), in accordance with the manufacturer’s instructions. The immobilized pre-mRNA beads were incubated at 30 °C for 20 min in 100 µL of HEK293 whole extract, RNase inhibitor (Takara Bio, Otsu, Japan), and nuclease-free water. Then, NET2 buffer (50 mM Tris-HCl (pH7.5), 150 mM NaCl, and 0.05% Nonidet P-40) was added to a final volume of 800 µL and incubated at 4 °C for 1 h. The incubated beads were washed five times with cold NET2 buffer and then boiled in 1× SDS-PAGE sample buffer for analysis by Western blotting.

### 4.7. Phosphatase Treatment

HeLa S3 cells were transfected with Rbm38-Myc plasmids using Lipofectamine 3000 reagent (Thermo Fisher Scientific). At 48 h post-transfection, cells were suspended in lysis buffer (25 mM HEPES, pH 7.5, 150 mM NaCl, 2 mM MgCl_2_, 1 mM EGTA, 1 mM EGTA, 1% Nonidet P-40, 10% glycerol, and cOmplete ULTRA tablets mini EDTA-free (Roche, Basel, Switzerland)), sonicated for 15 s, and centrifuged (13,000× *g*, 10 min, 4 °C) to remove debris. The lysates were treated with Lambda protein phosphatase (New England Biolabs, Ipswich, MA, USA), in accordance with the manufacturer’s instructions, and analysed by Western blotting.

### 4.8. Immunofluorescence

Cells were cultured on coverslips, fixed by adding formaldehyde (final concentration: 3.7%) directly to the cell culture medium, and then permeabilized with 0.5% Triton X-100 in PBS for 5 min. We incubated the coverslips with anti-Myc antibody at 4 °C overnight and then with the Alexa 488-conjugated anti-mouse secondary antibody at room temperature for 1 h. Cells were finally mounted in ProLong^®^ Gold Antifade Mountant with DAPI (Thermo Fisher Scientific) and observed under an IX73 microscope (Olympus, Tokyo, Japan).

### 4.9. Statistical Analysis

Statistical analysis was performed using R Commander. One-way ANOVA with Tukey’s multiple comparison test was used to determine statistical significance. Data are presented as the mean ± SD. The sample size in each experiment is mentioned in the figure legends. *p* < 0.05 was considered statistically significant.

## Supplementary Materials

Supplementary Materials can be found at https://www.mdpi.com/1422-0067/21/22/8799/s1.

## Abbreviations

Rbm38	RNA binding motif protein 38
Rbm24	RNA binding motif protein 24
SMEK2	Suppressor of MEK1 homolog 2
CDK6	Cyclin dependent kinase 6
EGFR	Epidermal growth factor receptor
VEGFA	Vascular endothelial growth factor A
RNP	Ribonucleoprotein
