# Supplementary material for: Rbm38 Reduces the Transcription Elongation Defect of the SMEK2 Gene Caused by Splicing Deficiency
Source: Int J Mol Sci. 2020 Nov 20;21(22):8799. doi: 10.3390/ijms21228799 (PMC7699959; doi:10.3390/ijms21228799)
Supplement: Supplementary file 1 [file ijms-21-08799-s001.pdf]

Supplementary Information

Rbm38 reduces transcription elongation defect of SMEK2 gene caused by splicing deficiency

Shintaro Muraoka[1], Kazuhiro Fukumura[2], Megumi Hayashi[1], Naoyuki Kataoka[3], Akila Mayeda[2] and Daisuke Kaida[1]*

[1] Graduate School of Medicine and Pharmaceutical Sciences, University of Toyama, 2630 Sugitani, Toyama 930-0194, Japan; kaida@med.u-toyama.ac.jp
[2] Division of Gene Expression Mechanism, Institute for Comprehensive Medical Science, Fujita Health University, Kutsukake-cho, Toyoake, Aichi 470-1192, Japan
[3] Department of Animal Resource Sciences, Graduate School of Agricultural and Life Sciences, The University of Tokyo, 1-1-1 Yayoi, Bunkyo-ku, Tokyo 113-8657, Japan

* Correspondence: kaida@med.u-toyama.ac.jp

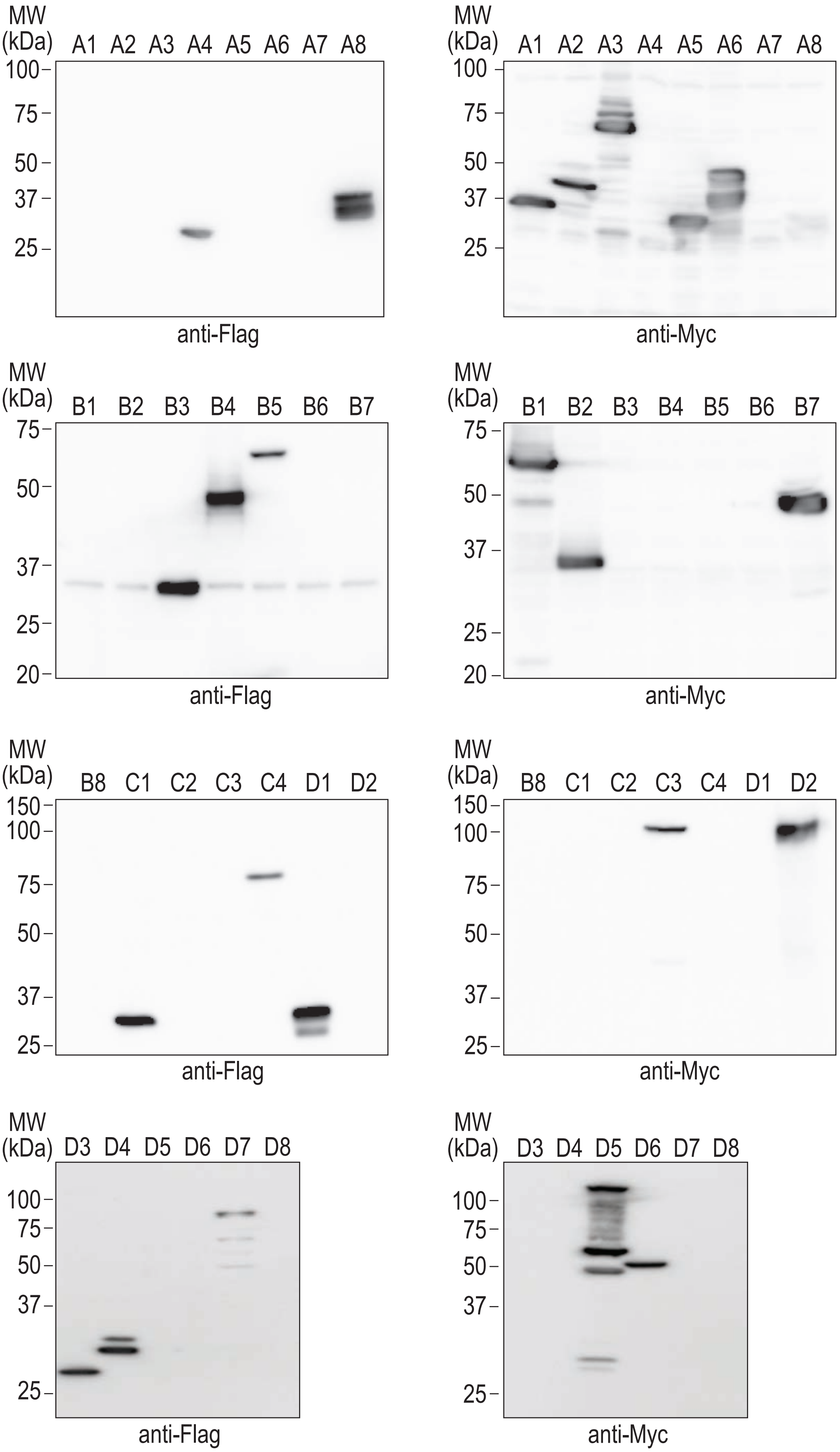

Figure S1. Protein expression of RNA-binding proteins.

HeLa cells were transfected with plasmids expressing RNA-binding proteins and then cultured for 48 h. Total lysate of the cells was analysed by western blotting to evaluate the expression levels of the RNA binding proteins.

Fig. S1 Muraoka et al.

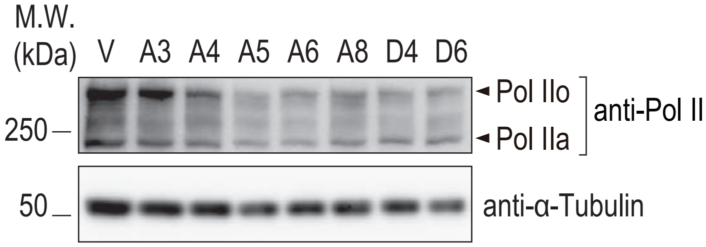

Figure S2. Phosphorylation status of Pol II upon over expression of RBPs.

HeLa cells were transfected with plasmids expressing RNA-binding proteins and then cultured for 48 h, then treated with 10 ng/ml SSA for 3 h. Total lysate of the cells was analysed by western blotting to evaluate the phosphorylation status of Pol II. Pol IIo: hyperphosphorylated form; Pol IIa: hypophosphorylated form.

Fig. S2 Muraoka et al.

```
  1 aaatgtgtgtgtaggctgggtgcagtggctcacacctgtaatcccagcac 50
 51 tttgggaggtcgaggcaggcggatcgcttgagcccaggagttggagttcc 100
101 agaccaacctgggcaaaatggtgaaaccctgtctctacgaaaaattagtg 150
151 tggcgtggtgtgtgcacctgtagtcccagctacttgggaggctgaggtgg 200
201 gaggattgctggggcctaggaggtcgaggctgcagtgagtcgtgattgta 250
251 cccctacactctagtctggtcacagagcaagaccctgtctcaaggaggaa 300
301 acaacaaaatatatatatgtatatttgtgtgtatgtgtgtgtgtgtgtgt 350
351 gtgtctatgttttattttaaatccaaaccttggtttctaatacaaggggt 400
401 gacaaccttttcctaaaaatgtaactaaccctttggtaggcatataattg 450
451 aagacattctaggagactgcttacctaaaccttgggctaatttttctaca 500
501 ccctaagctaaggtactgaactgggtgc
```

Figure S3. The DNA sequence of the part of the *SMEK2* intron 4 used for the RNA binding assay.

The red letters indicate putative Rbm38 binding sequences.

Fig. S3 Muraoka et al.

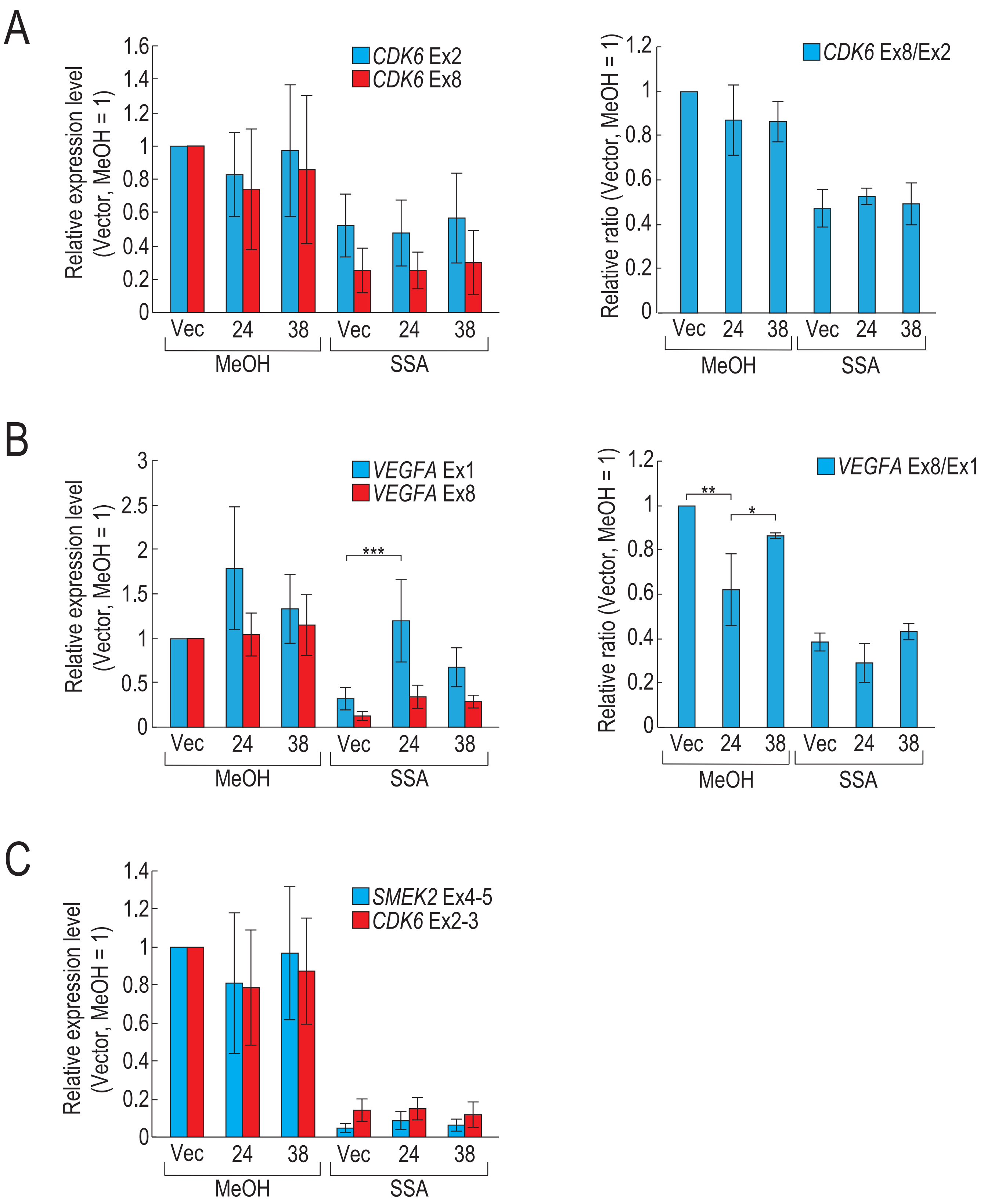

Figure S4. The effects of Rbm38 and Rbm24 on transcription elongation and splicing.

(A, B, C) HeLa cells were transfected with a plasmid, Flag-Rbm24 or Flag-Rbm38, and then cultured for 48 h. The transfected cells were treated with SSA and 5-EU, and the labelled RNA was analysed as in Fig. 1. Error bars indicate S.D. (n = 3). Statistical significance was investigated by one-way ANOVA and Tukey' s test (*: $p<0.05$; **: $p<0.01$; ***: $p<0.001$).

Fig. S4 Muraoka et al.

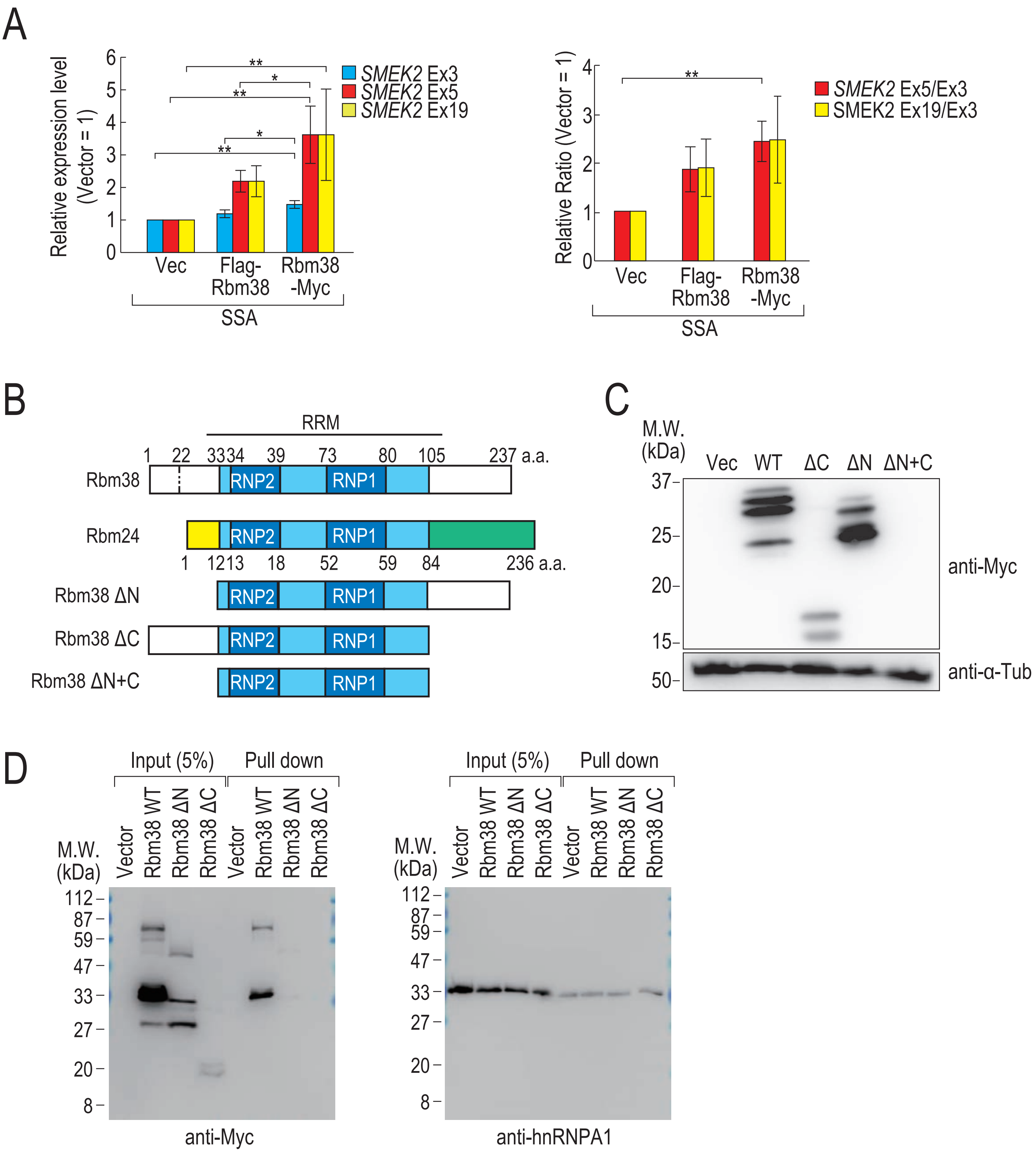

Figure S5. The N- and C-terminal regions are important for gene expression and RNA binding capacity. (A) HeLa cells were transfected with a plasmid, Flag-Rbm38 or Rbm38-Myc, and then cultured for 48 h. The transfected cells were treated with SSA and 5-EU, and the labelled RNA was analysed as in Fig. 1. Error bars indicate S.D. (n = 3). Statistical significance was investigated by one-way ANOVA and Tukey' s test (*: $p<0.05$; **: $p<0.01$). (B) A schematic of the structure of the indicated RNA binding proteins. (C) HeLa cells were transfected with a vector (Vec), Rbm38-WT or the N- and/or C-terminal region deletion mutants (ΔN, ΔC, ΔN+C), and then cultured for 48 h after transfection. Protein samples were prepared and analysed by western blotting. (D) A biotinylated RNA pull-down assay was performed using WT and the N- and/or C-terminal region deletion mutants as in Fig. 2C.

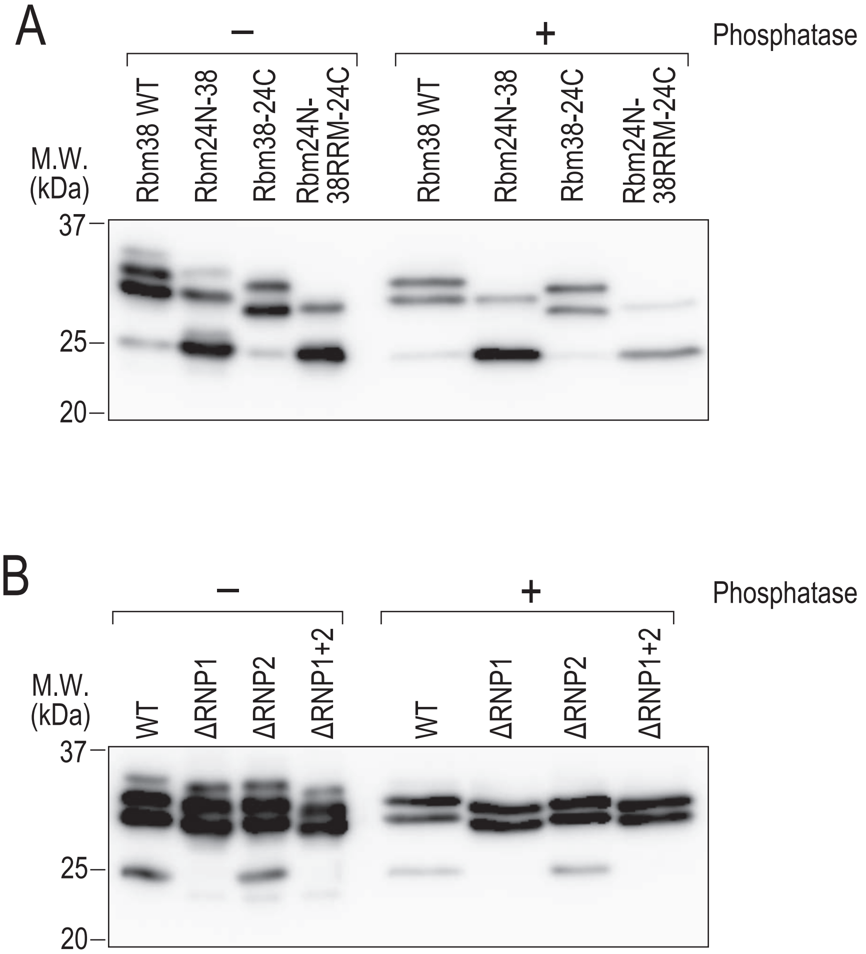

Figure S6. Exogenous Rbm38 WT and mutants are phosphorylated and truncated.

(A, B) HeLa cells were transfected with Rbm38 plasmids and cultured for 48 h, Total lysate were prepared and treated with protein phosphatase. The lysates were analysed by western blotting.

Fig. S6 Muraoka et al.

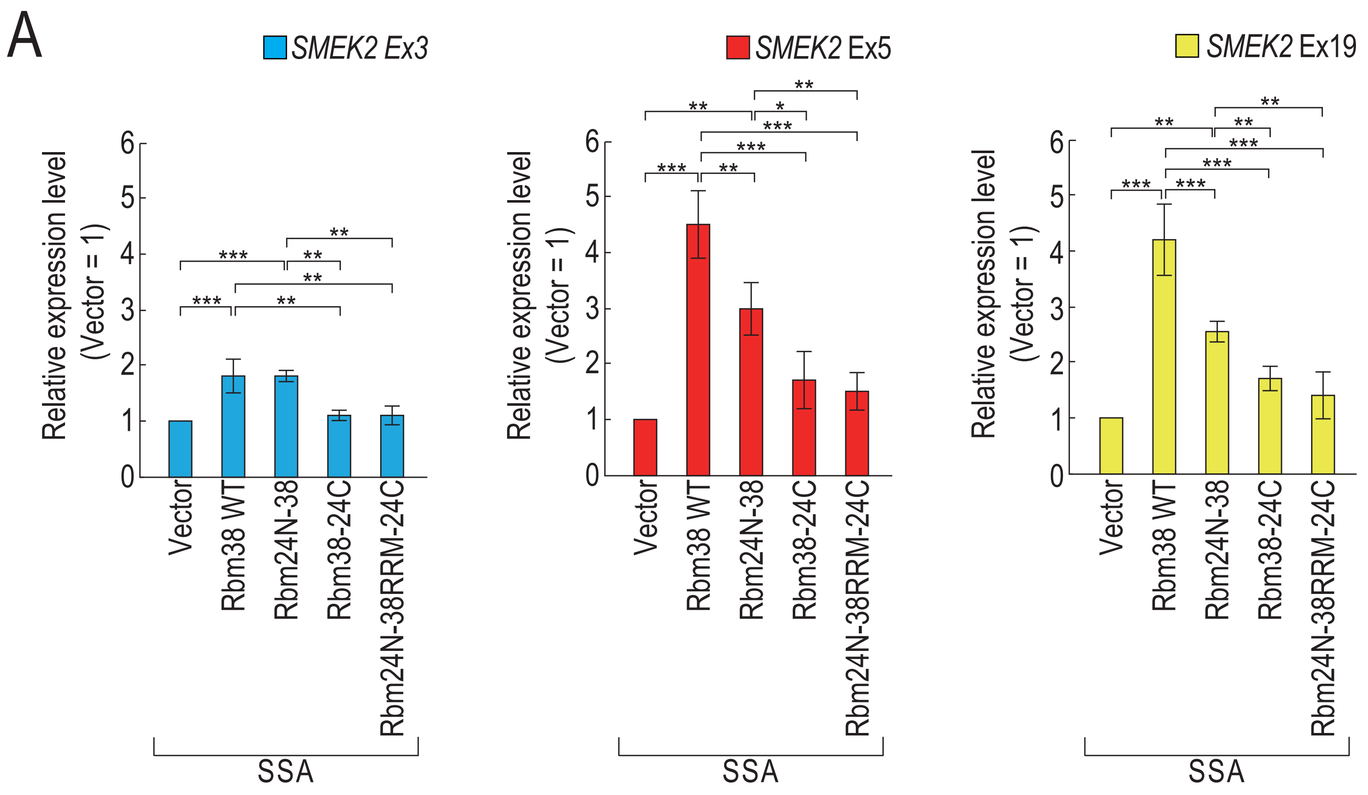

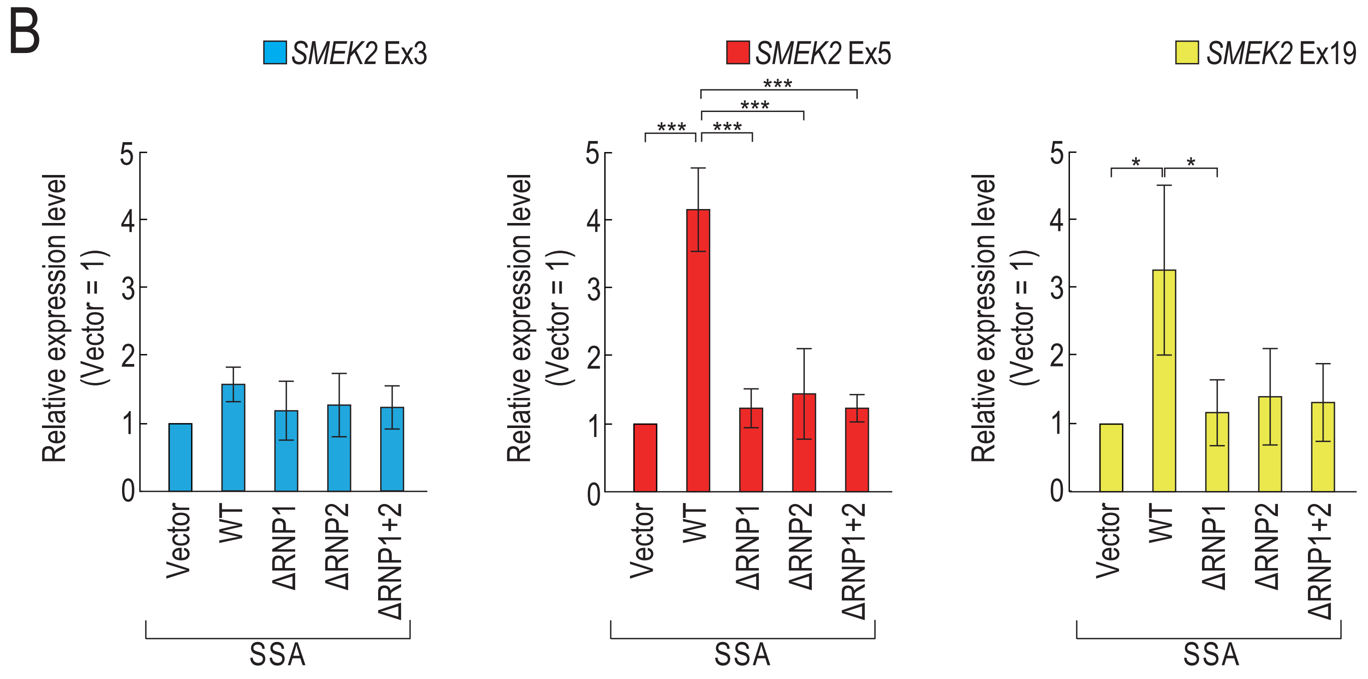

Figure S7. The N- and C-terminal regions and RBD/RRM domain of Rbm38 are important for suppressing the transcription elongation defect.

(A, B) HeLa cells were transfected with a vector or chimeric plasmids, and then cultured for 48 h after transfection (A). HeLa cells were transfected with a vector or RNP deletion mutants, and then cultured for 48 h (B). The transfected cells were treated with SSA and 5-EU, and the labelled RNA was analysed as in Fig. 1. Error bars indicate S.D. (n = 3). Statistical significance was investigated by one-way ANOVA and Tukey' s test (*: p<0.05; **: p<0.01; ***: p<0.001).

Fig. S7 Muraoka et al.

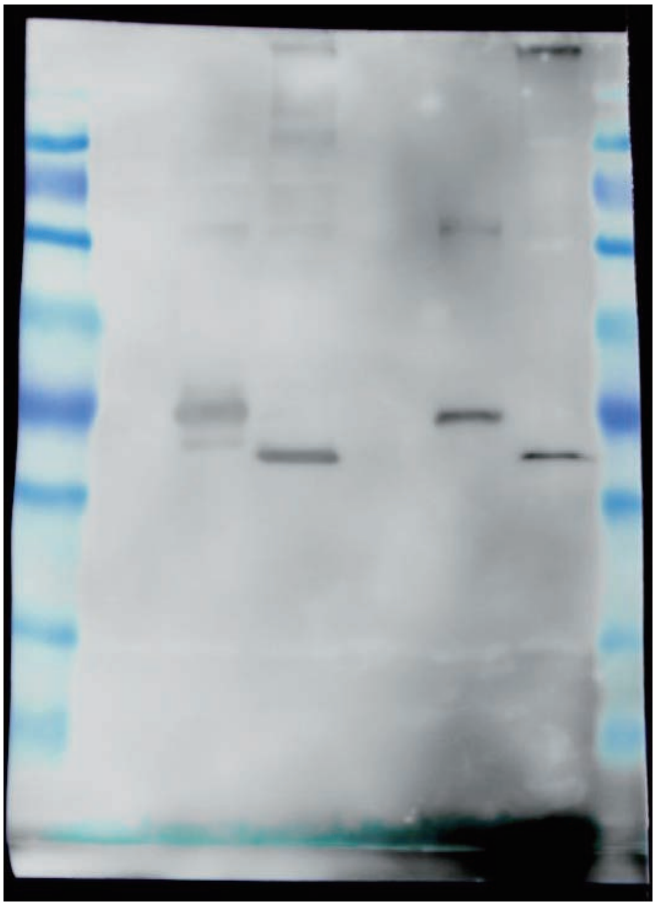

anti-Flag

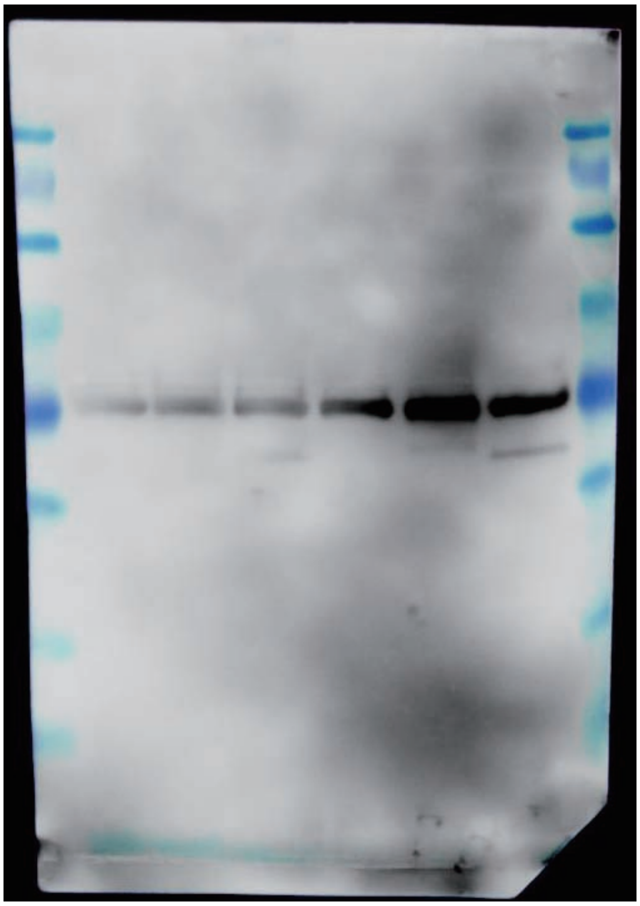

anti-hnRNPA1

Figure. S8. Images of the full-length, unprocessed membranes and western blots shown in Fig. 2C.

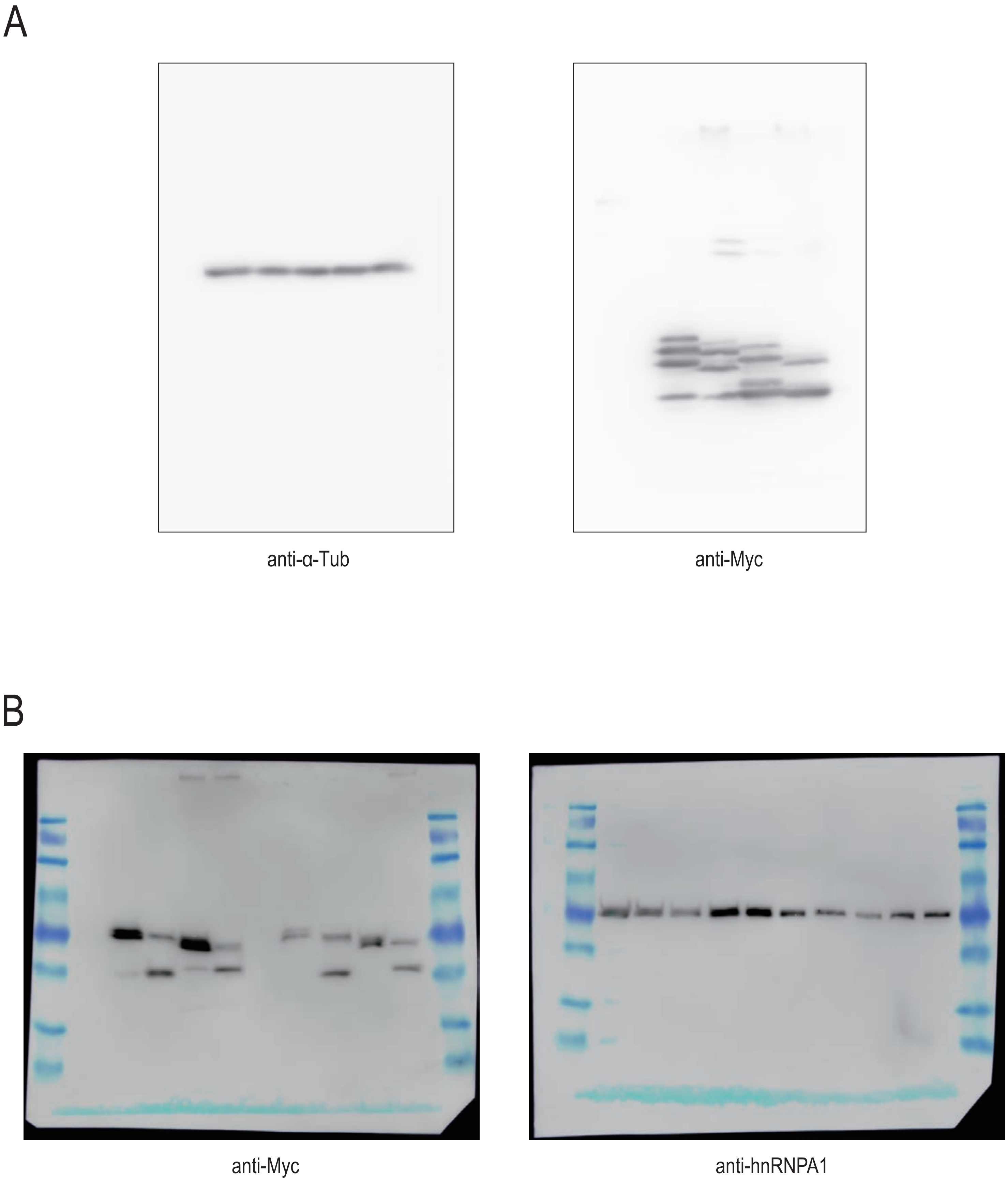

Figure. S9. Images of the full-length, unprocessed membranes and western blots shown in Fig. 3B (A) and 3D (B).

A

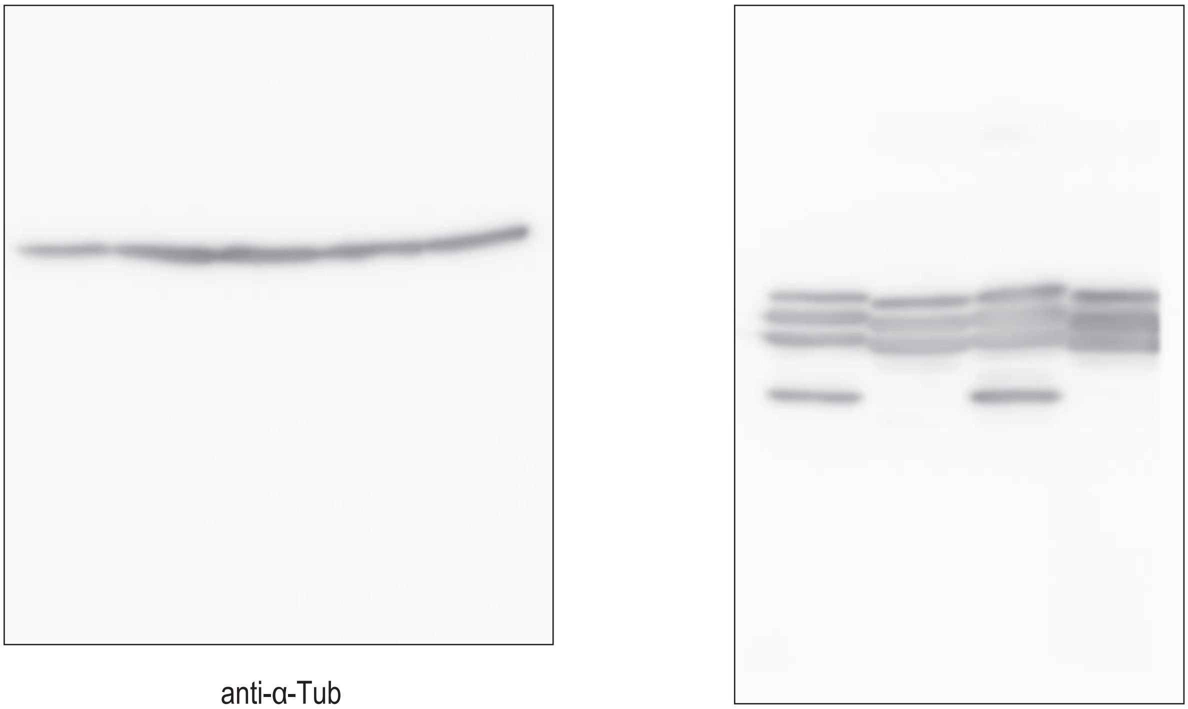

anti-α-Tub

anti-Myc

B

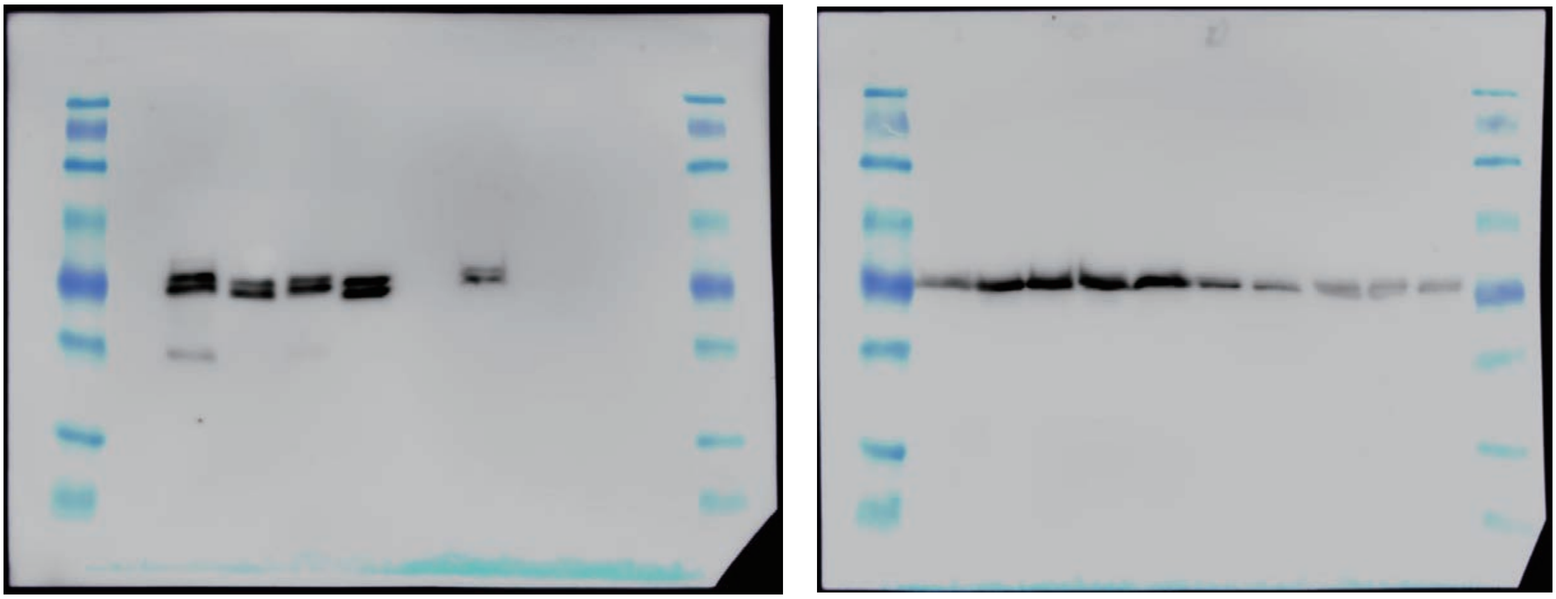

anti-Myc

anti-hnRNPA1

Figure. S10 Images of the full-length, unprocessed western blots shown in Fig. 4B (A) and 4D (B).

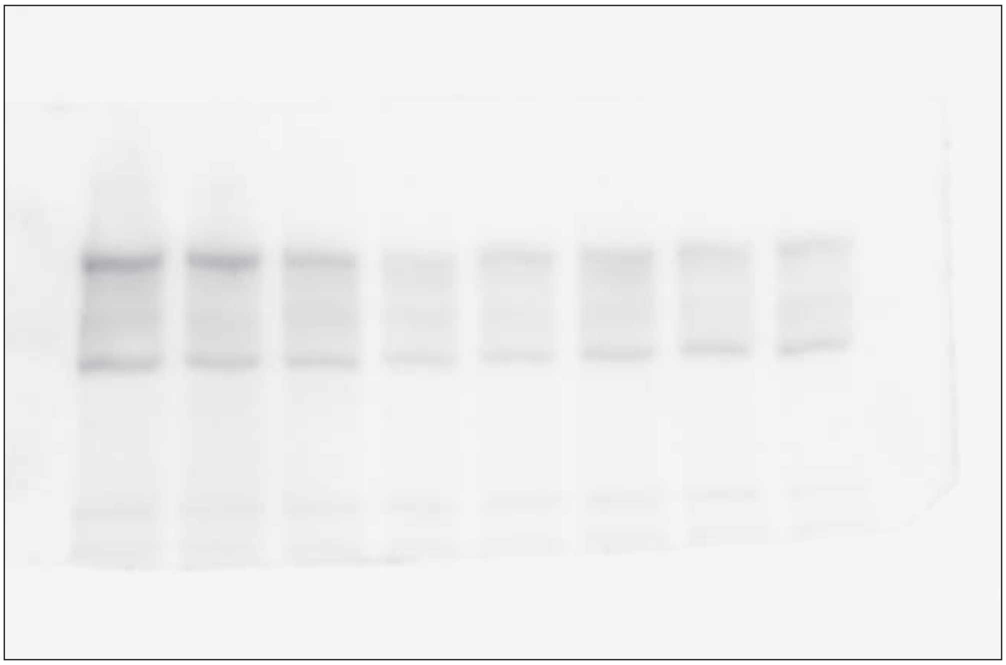

anti-Pol II

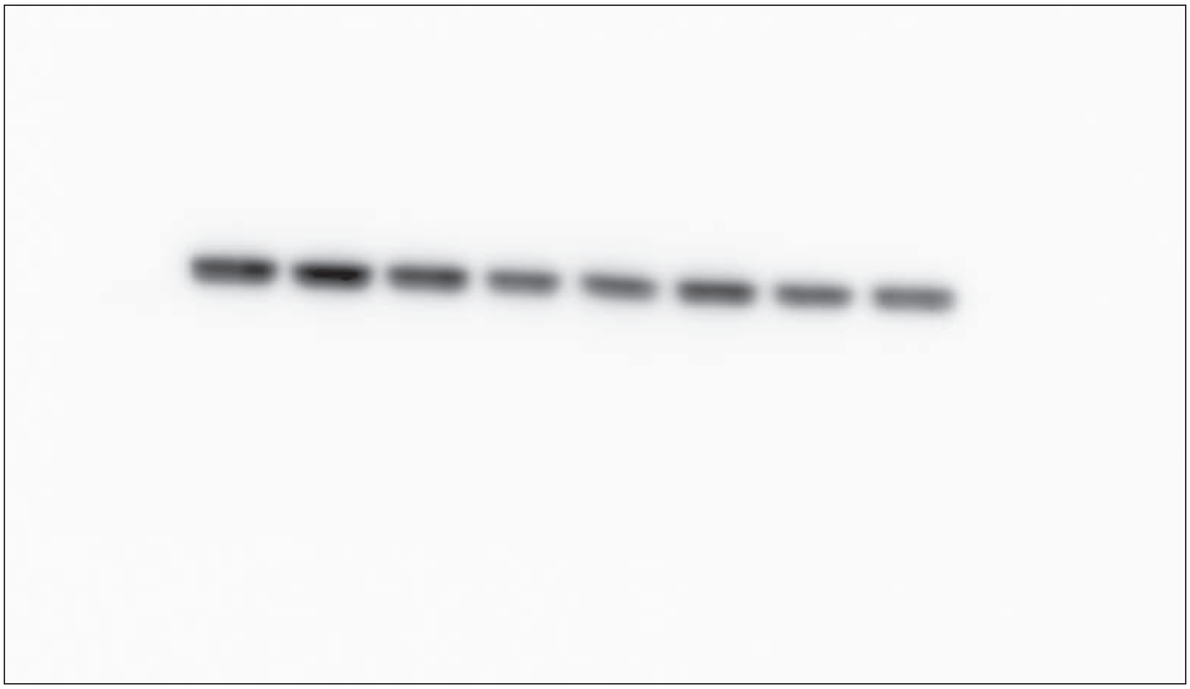

anti-α-Tubulin

Figure. S11. Images of the full-length, unprocessed western blots shown in Fig. S2.

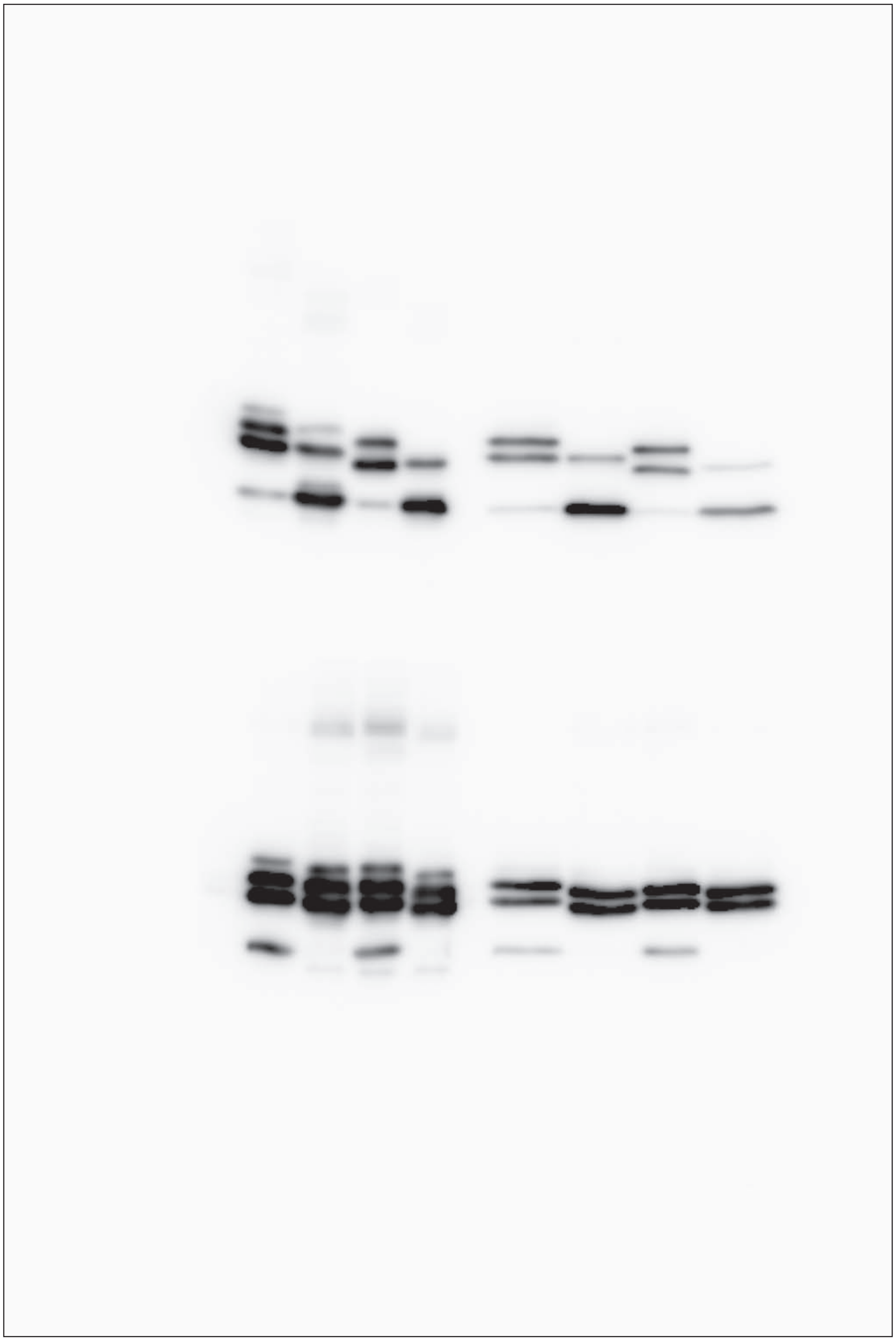

Figure. S12. Images of the full-length, unprocessed western blots shown in Fig. S6.

Table S1 RNA binding proteins used in this study. The 23 RBPs which were used in Figure 1 were in red letters.

A1	Myc-hnRNP A
A2	Myc-hnRNP C
A3	Myc-hnRNP L
A4	Flag-SRp30 (SRSF9)
A5	Myc-SC35 (SRSF2)
A6	Myc-SRp46 (SRSF8)
A7	Myc-9G8 (SRSF7)
A8	Flag-mouseSF2 (SRSF1)
B1	Myc-U2AF65
B2	Myc-U2AF35
B3	Flag-Aly
B4	Flag-RNPS1
B5	Flag-hUpf3
B6	Flag-Magoh
B7	Myc-eIF4AIII
B8	Flag-MLN51
C1	Flag-Y14
C2	Flag-DEK
C3	Myc-CBP80
C4	Flag-RBM17
D1	Flag-SRp20
D2	Myc-SRp75
D3	Flag-mouseRBM24
D4	Flag-mouseRBM38
D5	Myc-PUF60
D6	Flag-mouseCUGBP2
D7	PSF/SFPQ-Flag
D8	Flag-Staufen

Table S2 Top10 Rbm38 binding motifs reported by Heinicke et al. (Ref. 22)

GTGTGTG
TGTGTGT
GTGTGTT
GGTGTGT
AGTGTGT
CGTGTGT
TTGTGTG
GTTGTGT
GTGTGGT
TGGTGTG

Table S3. List of primers used in this study.

For cloning	
Name	Sequence
mRBM38F	CCCGAATTCATGCTGCTGCAGCCCG
mRBM38R	CCCCTCGAGCTGCATCCTGTCAGGCTGTAG
RBM38ΔNF	CCCGAATTCATGACCAAGATCTTCGTGGGCG
RBM38ΔCR	CCCCTCGAGCAGGTAGGCCAGGTTCACATTG
Chimera1F	CGAATTCATGCACACCACCCAGAAGGACACGACGTAC ACCAAGATCTTCGTGGGCGGCCT
Chimera2R	TGCTCCCAAGTATGCCAGGTTCACATTGGCCTTGCG
Chimera3F	CGCAAGGCCAATGTGAACCTGGCATACTTGGGAGCA
mRBM24R	CCCCTCGAGCTGCATTCGGTCTGTCTGC
ΔRNP1F	CGGCAAGTCCGCAGATCGGGCAGCGGC
ΔRNP1R	CCCGATCTGCGGACTTGCCGGTCTGGC
ΔRNP2F	GTTCACCAAGCCCTACCACACCACCGAC
ΔRNP2R	TGTGGTAGGGCTTGGTGAACGTGGTGTC
SMEK2 int4 cloning for EcoRI	GCCGCCGAATTCAAATGTGTGTGTAGGCTGGGTG
SMEK2 int4 cloning rev XhoI-3	GCCGCCCTCGAGGCACCCAGTTCAGTACCTTAGC
For RT-qPCR	
Name	Sequence
18S rRNA for	GTTGGTGGAGCGATTTGTCTGGTT
18S rRNA rev	TATTGCTCAATCTCGGGTGGCTGA
SMEK2 Ex3 for	TGCCTTTACCGTCTCCTAAGAGTG
SMEK2 Ex3 rev	ATTGCCGGTCTTCGTTCAGGGTAT
SMEK2 Ex5 for	TGTTTGGTCAGAAGCAGAGAA
SMEK2 Ex5 rev	TTTCCCAGATCTCATCACAGC
SMEK2 Ex19 for	GGAAGTTTGGTTGGCTTAGTGG
SMEK2 Ex19 rev	GCTCACTGAACAGTTGCAGCATTG
CDK6 Ex2 for	AGTACGAATGCGTGGCGGAGAT
CDK6 Ex2 rev	AAACGGCCTCCGTTCTTCAAGT
CDK6 Ex8 for	CTGCTGACCAATTGTGCTGCCATT
CDK6 Ex8 rev	CACACACACATGCACACACACACT
VEGF Ex1 for	GAGGCGCAGCGGTTAGGT
VEGF Ex1 rev	CGGATCAATGAATATCAAATTCCA
VEGF Ex8 for	CTGGCGCTGAGCCTCTCTAC
VEGF Ex8 rev	CCGGTGTCCTCATCCCTGTA
CDK6 Ex2-3 spliced for	AGGCACCTGGAGACCTT
CDK6 Ex2-3 spliced rev	TGGTTTCTCTGTCTGTTCGTG
SMEK2 Ex4-5 unspliced for	CCCTATTCTACAGTTCCGATAACC
SMEK2 Ex4-5 unspliced rev	AGTTCTCTGCTTCTGACCAAAC

Table S4. List of plasmids used in this study.

Name	
Rbm38 WT	Myc-tagged Rbm38
Rbm38 ΔN	N-terminal deletion mutant of Rbm38
Rbm38 ΔC	C-terminal deletion mutant of Rbm38
Rbm38 ΔN+C	N- and C-terminal deletion mutant of Rbm38
Rbm24N-38	N-terminal region of Rbm38 is replaced by that of Rbm24
Rbm38-24C	C-terminal region of Rbm38 is replaced by that of Rbm24
Rbm24N-38RRM-24C	Both N- and C-terminal regions of Rbm38 are replaced by those of Rbm24
Rbm38 ΔRNP1	RNP1 deletion mutant of Rbm38
Rbm38 ΔRNP2	RNP2 deletion mutant of Rbm38
Rbm38 ΔRNP1+2	RNP1 and RNP2 deletion mutant of Rbm38
